# A New Strategy for Ultrasensitive Detection Based on Target microRNA-Triggered Rolling Circle Amplification in the Early Diagnosis of Alzheimer’s Disease

**DOI:** 10.3390/ijms25179490

**Published:** 2024-08-31

**Authors:** Fei Zhao, Na Zhang, Yi Zhang

**Affiliations:** 1 Academy of Medical Engineering and Translational Medicine, Tianjin University, 92 Weijin Road, Nankai District, Tianjin 300072, China; 2 Tianjin Key Laboratory of Brain Science and Neural Engineering, Tianjin University, 92 Weijin Road, Nankai District, Tianjin 300072, China

**Keywords:** Alzheimer’s disease, plasma miRNAs, biomarkers, rolling circle amplification, early diagnosis

## Abstract

There is an urgent need to accurately quantify microRNA (miRNA)-based Alzheimer’s disease (AD) biomarkers, which have emerged as promising diagnostic biomarkers. In this study, we present a rapid and universal approach to establishing a target miRNA-triggered rolling circle amplification (RCA) detection strategy, which achieves ultrasensitive detection of several targets, including miR-let7a-5p, miR-34a-5p, miR-206-3p, miR-9-5p, miR-132-3p, miR-146a-5p, and miR-21-5p. Herein, the padlock probe contains three repeated signal strand binding regions and a target miRNA-specific region. The target miRNA-specific region captures miRNA, and then the padlock probe is circularized with the addition of T4 DNA ligase. Subsequently, an RCA reaction is triggered, and RCA products containing multiple signal strand binding regions are generated to trap abundant fluorescein-labeled signal strands. The addition of exonuclease III (Exo III) causes signal strand digestion and leads to RCA product recycling and liberation of fluorescein. Ultimately, graphene oxide (GO) does not absorb the liberated fluorescein because of poor mutual interaction. This method exhibited high specificity, sensitivity, repeatability, and stability toward let-7a, with a detection limit of 19.35 fM and a linear range of 50 fM to 5 nM. Moreover, it showed excellent applicability for recovering miRNAs in normal human serum. Our strategy was applied to detect miRNAs in the plasma of APP/PS1 mice, demonstrating its potential in the diagnosis of miRNA-associated disease and biochemical research.

## 1. Introduction

Alzheimer’s disease (AD) is a progressive, irreversible neurodegenerative disorder that has significant economic and societal impacts; its global cases will triple by 2050 [1]. Unfortunately, the lack of effective diagnostic tools for real-time probing of initial phases of AD may lead to the failure of AD drug development. Current drugs for the treatment of AD only delay disease onset and progression [2]. Therefore, it is critical to develop reliable diagnosis methods and identify novel biomarkers for AD, ideally during the early stages. AD is caused by multiple interactions between genetic and environmental factors, and various pathologies co-occur with AD, including cardiovascular disease, hypertension, diabetes, Parkinson’s disease, and other diseases; the coexistence of these multiple diseases makes the search for AD biomarkers more challenging [3,4,5,6]. In recent years, blood-based biomarkers for early AD diagnosis have gained attention due to their non-invasiveness, and various miRNAs are differentially expressed in AD patients and healthy individuals, which makes blood-based miRNA biomarkers an attractive approach [7,8,9,10]. However, the alteration of miRNA expression during the disease process often leads to conflicting results in many studies on AD-related miRNAs [4,11,12]. In addition, due to the complex gene-miRNA regulatory network in AD, miRNA-based signatures of AD might prove to be useful as biomarkers for AD [13]. Therefore, simultaneous detection of multiple biomarkers is an effective means to improve the accuracy of AD diagnosis [14,15].

Standard analytical approaches for quantifying miRNAs include northern blotting, quantitative reverse transcription polymerase chain reaction (qRT-PCR), next-generation sequencing, and microarrays. Among these methods, only northern blotting can directly detect native miRNAs, while other methods require additional labeling or amplification steps [16,17,18]. Furthermore, some of these techniques require special equipment and professionally trained operators to handle complicated and time-consuming procedures [19]. Alternatively, recent advances in miRNA biosensing techniques, such as RCA, as one of the isothermal amplification technologies, has shown great promise because of simple operation and high sensitivity that overcome the limitations of conventional methods [20,21,22,23]. RCA-based amplification technology achieves performance optimization by combining with other technologies. For example, CRISPR-Cas9 is used to recognize and cleave target sequences to activate RCA reactions [24], RCA products are designed as G-quadruplex structures for detection by binding with hemin [22], and when RCA products hybridize with the second or even third primer, hyperbranched RCA will be initiated for further amplification in a short time [25]. When RCA is combined with nucleic acid endonucleases, multiple primer copy sequences will be generated, and the reaction will enter exponential amplification [26]. In summary, RCA technology is very suitable for detecting low-abundance miRNAs in biological fluids.

Graphene oxide (GO) is one typical two-dimensionally structured and oxygenated planar molecular material with a large surface area and high absorption over single-stranded DNA (ssDNA), making it an excellent quencher [27,28]. GO possesses a large capacity for loading ssDNA via hydrogen bonds and pi stacking interactions between aromatic ring structure of ssDNA bases and the GO surface. Thus far, ssDNA-labeled fluorophore can potentially form a good donor–acceptor pair with GO through long-range energy transfer. Due to these properties, we recently used GO as a fluorescence quenching material for distinguishing fluorescently labeled single-stranded oligonucleotides and double-stranded nucleic acids. The GO-based fluorescence sensor could determine the concentration of target genes by the quenching and recovery of fluorescence, which has been used to detect various biological markers, such as DNA [29,30], miRNA [31,32], and proteins [33,34]. In this study, fluorescently labeled DNA was used as an oligonucleotide probe, and GO was used as a fluorescence quencher to sensitively detect single-stranded RCA products (RCAPs) containing amplified miRNA sequences.

In this study, a method for ultrasensitive detection of miRNAs was established by combining the identification of target miRNAs, RCA, Exo III enzyme-assisted cyclic amplification, and a GO-based fluorescence quenching system, which was named Exo III-GO-RCA. This method was triggered by target miRNAs, and a padlock sequence with a triple repetitive signaling strand-binding region was used as a template for RCA detection, which detected the fluorescence signal originating from the free fluorescein released after the Exo III cycle sheared the double strand and quenched the false-positive signal with GO. The limit of detection (LOD) for let-7a was 19.35 fM, and the linear working range was 50 fM–5 nM. The assay system had no cross-reactivity with non-target let-7c, let-7b, miR-455, and miR-34c, and it had the ability of single-base recognition; in the case where the concentration of non-target miR-34c was 1000-fold higher than that of the target, the target could be distinguished and the system was highly resistant to interference. It could be used for the detection of complex biological samples, with recoveries ranging from 95% to 102.4%. Unlike conventional PCR assays that require a thermal cycler and reverse transcription, this assay platform can amplify at lower and constant temperatures, and its linear range for let-7a detection at low concentrations was comparable to that of qRT-PCR research methods. This method was used to test for differential miRNA expression during early AD, and we assessed plasma samples from C57BL/6J controls (WT, male: *n* = 6, female: *n* = 6) and APP/PS1 mice (male: *n* = 6, female: *n* = 6) to verify significant differences between miR-34c-5p, miR-206-3p, miR-9-5p, miR-132-3p, miR-146a-5p, and miR-21-5p expression in the WT and AD groups. Combining the changes in six different miRNAs’ expression, we demonstrated that miR-34c, miR-206, and miR-9-5p yielded superior diagnostic performance for female AD. Consequently, our research suggests the remarkable potential of developing an early AD screening platform based on the expression of plasma AD miRNA biomarkers.

## 2. Results 

### 2.1. Principle of Exo III-GO-RCA Assay

**Figure 1:**
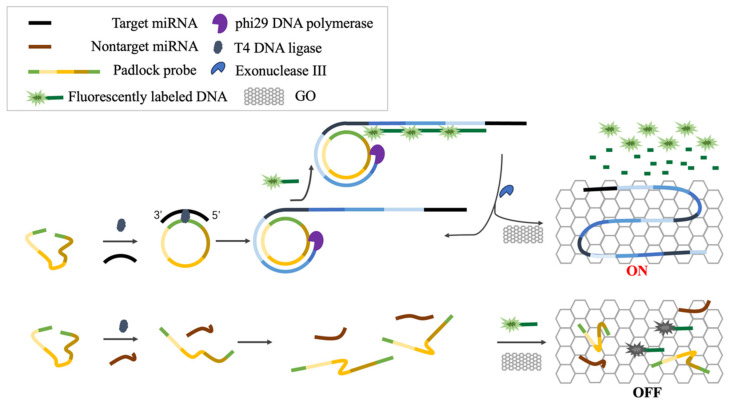
Schematic illustration of the Exo III-GO-RCA strategy for miRNA detection.

The miRNA intended for detection is amplified using Exo III-GO-RCA (Figure 1). The padlock probe is composed of a hybridization sequence to miRNA and three-segmented repetitive signal probe recognition regions. The 5′-PO3 group of the padlock probe is adjacent to its 3′-OH group, which allows a phosphodiester linkage to be formed with the help of T4 DNA ligase to form a mixed closed ring structure. The circular DNA serves as the RCA template, and the target miRNA becomes the primer, which will be extended to synthesize a long single-stranded DNA sequence catalyzed by phi29 DNA polymerase. However, in the absence of target miRNA, circular DNA formation and synthesis of long single-stranded DNA do not take place. The RCA product synthesized in the presence of the target miRNA forms a double strand by binding to the fluorescein-labeled signaling probe, and the double-stranded structure releases fluorescein and the RCA product by digestion with Exo III, which then proceeds to the next step in the cycle of signaling probe binding and fluorescein release [31]. The released fluorescein has little interaction force with GO and the fluorescence is not quenched. When the target miRNA is not present and its corresponding RCA product is not formed, the free single-stranded fluorescent signaling probe will be adsorbed on the GO monolayer and the fluorescence is quenched. Therefore, it is possible to quantitatively detect miRNAs by simply measuring the fluorescence signal after adding GO to the reaction mixture.

### 2.2. Assay Feasibility

**Figure 2:**
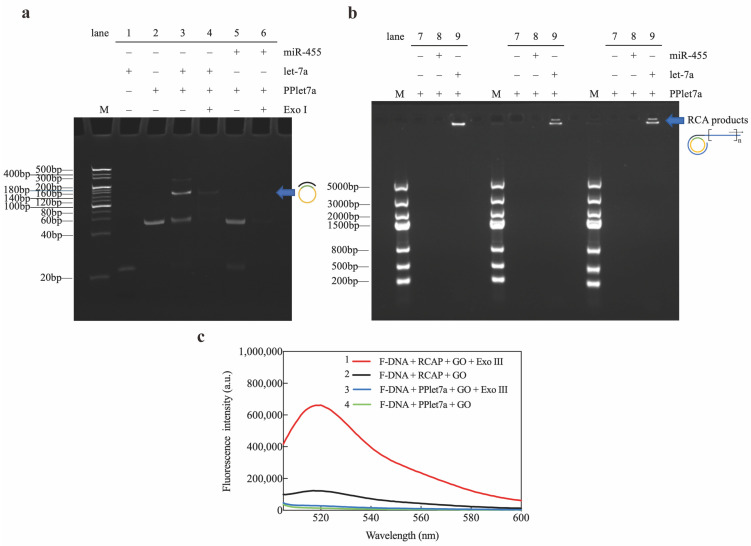
Validation of the Exo III-GO-RCA assay: (**a**) Non-denaturing PAGE assay of circularization products produced under different conditions; (**b**) Agarose gel electrophoresis assay of let-7a-triggered RCA reaction system. The experimental conditions for lanes 1–9 are shown at the top of the figures; (**c**) Fluorescence emission spectra (excitation/emission: 495/519 nm) under different conditions using the let-7a-triggered Exo III-GO-RCA reaction system.

The feasibility of this fluorescent assay platform for miRNA detection was determined using electrophoretic analysis and fluorescence quenching. We performed ligation and RCA in the presence of target let-7a or non-target miR-455 to examine whether the target miRNA sequence was specifically amplified through ligation and subsequent RCA. The two ends of the padlock sequence (Figure 2a, lane 2) were hybridized with the bases of the connection let-7a sequence (Figure 2a, lane 1), and the end segments of the padlock sequence were connected by the ligation reaction, forming the circular DNA (Figure 2a, lane 3). By contrast, when PPlet-7a was mixed with miR-455, only a single PPlet-7a band was observed (Figure 2a, lane 5). Exonuclease I (Exo I) was used to check the formation of the circular DNA, which digested ssDNA in a 3′-to-5′ direction but did not digest the double-stranded or circular DNA. The padlock probe was degraded upon the addition of Exo I to the ligation reaction mixture (Figure 2a, lane 6), and the band corresponding to the circular DNA remained intact (Figure 2a, lane 4). This indicated that circularization of the padlock probe specifically occurred in the presence of the target miRNA.

Next, it was verified that the target let-7a sequence could be specifically amplified by RCA. RCA was carried out in the presence of target let-7a or non-target miR-455. A large, high-molecular-weight DNA molecule was observed only in the presence of let-7a, suggesting that the RCAP generated by RCA was a long ssDNA containing possibly multiple copies –of the target let-7a sequence (Figure 2b, lane 9). By contrast, RCA product was not generated in the absence of let-7a (Figure 2b, lane 7) or in the presence of non-target miR-455 (Figure 2b, lane 8). These results suggested that the RCA reaction occurred only in the presence of padlock probes and corresponding miRNAs with complementary sequences.

To validate the fluorescence detection system of the method, this study monitored the fluorescence changes of FAM-labeled DNA (F-DNA) by adding RCA products, Exo III, and GO to the F-DNA mixture. When target let-7a was not added to the RCA reaction mixture, the fluorescence intensity of the mixture of its reaction product and F-DNA was weak at 519 nm (Figure 2(c4)), which indicated that if let-7a was not present, the RCA reaction would not be triggered and the corresponding amplification product would not be produced, and the signaling probe would not be able to form a double-stranded structure with the RCA product; thus, the fluorescence would be quenched significantly by GO. And if Exo III was added to the above solution, since F-DNA is a single-stranded structure, it could not be degraded by Exo III but was adsorbed on GO to quench the fluorescence, and the fluorescence intensity was still weak at this time (Figure 2(c3)). When let-7a was present in the RCA reaction mixture, the RCA reaction was triggered and a long DNA single strand was produced; at this time, the RCA product formed a double strand with F-DNA, the F-DNA fluorescence would not be quenched by GO, and at this time, the fluorescence intensity of the RCA system was significantly increased (Figure 2(c2)). When Exo III was then added to this system, Exo III degraded the fluorescent signaling probe complementary to the long RCA product, resulting in the release of fluorescein on the signaling probe while the long ssDNA product was recovered and reused to bind to other signaling probes, ultimately leading to further amplification of the fluorescence intensity (Figure 2(c1)). The above results illustrate the feasibility of signal amplification using this method and Exo III.

### 2.3. Optimization and Sensitivity of Exo III-GO-RCA Strategy

**Figure 3:**
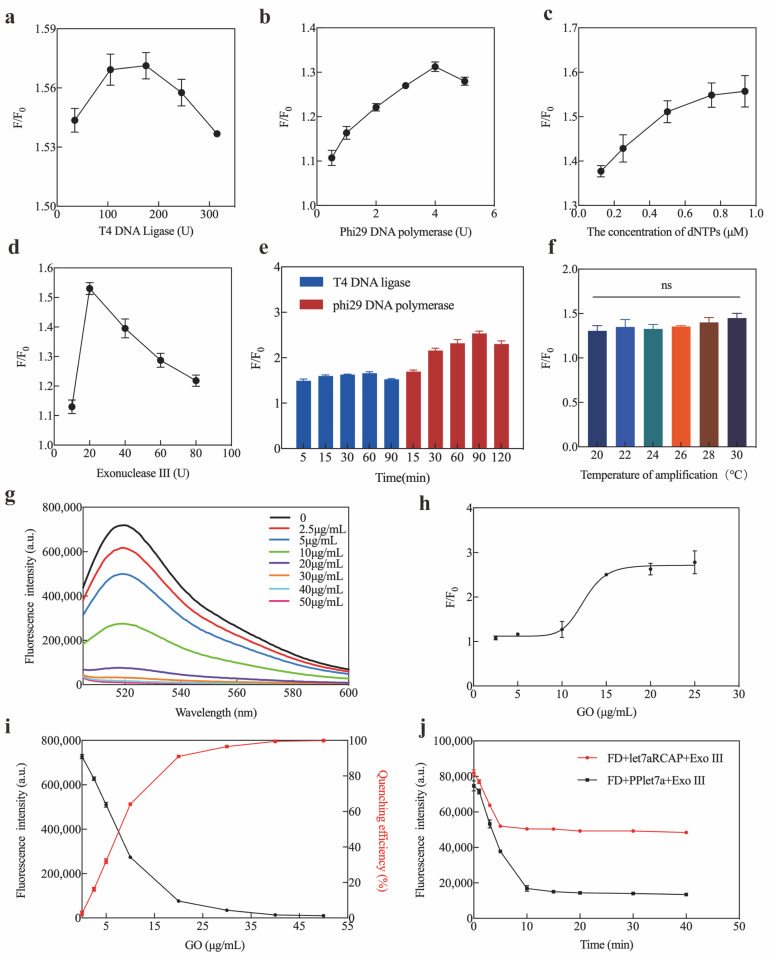
Optimization of the assay under different conditions: (**a**) Different amounts of T4 DNA ligase; (**b**) Different amounts of phi29 DNA polymerase; (**c**) Different concentrations of dNTPs; (**d**) Different amounts of Exo III; (**e**) Variations in reaction time; (**f**) Variations in RCA reaction time; (**g**–**i**) Fluorescence quenching of F-DNA in different concentrations of GO (0, 2.5, 5, 10, 20, 30, 40, and 50 μg/mL); (**j**) GO effect of time. F and F_0_ are the fluorescence intensity in the presence and absence of target let-7a, respectively. “ns” means no significant difference between groups.

**Figure 4:**
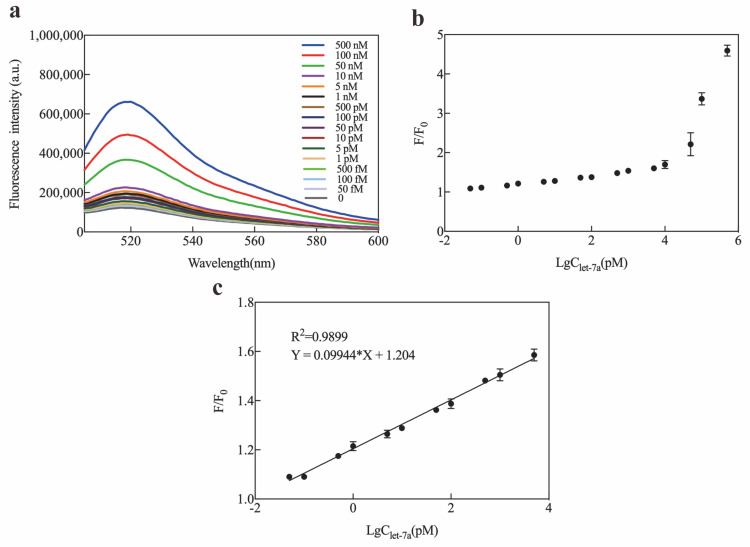
Sensitivity of the Exo III-GO-RCA assay: (**a**) Fluorescence emission spectra obtained from the RCAPlet7a/F-DNA hybrid in the presence of Exo III and GO upon addition of let-7a at different concentrations (0–500 nM). The maximal emission fluorescence at 519 nm was measured; (**b**) Relationship between Fluorescence intensity at 519 nm and logarithm of let-7a concentration from 50 fM to 500 nM; (**c**) Plot of fluorescence ratio (F/F_0_) of the system with the different concentrations of let-7a. Where F_0_ and F are the fluorescence intensities in the absence and presence of the microRNA let-7a, respectively.

To improve the sensitivity of the Exo III-GO-RCA strategy for let-7a detection, optimization experiments were performed, including T4 DNA ligase, phi29 DNA polymerase, dNTPs, Exo III, and GO concentrations. The fluorescence signal of the reaction system increased with increasing concentrations of T4 DNA ligase, Phi29 DNA polymerase, and dNTPs until the signal-to-noise ratio of the system was maximal when using T4 DNA ligase at 175 U, 4 U of Phi29 DNA polymerase, 0.75 μM of dNTPs, 1 U of Exo III, and 20 μg/mL of GO (Figure 3a–d). According to the optimization results, a ligation time of 60 min, an amplification time of 90 min, an amplification temperature in the room temperature range (20 to 30 degrees Celsius) (Figure 3e,f), and a GO quenching time of 10 min were the optimal Exo III-GO-RCA reaction conditions (Figure 3g–j). Under the best optimized conditions, the luminescence intensity of the Exo III-GO-RCA system increased consistently with increasing let-7a concentration (Figure 4a). Figure 4b shows the scatter plot of the signal-to-noise ratio of the fluorescence intensity of the reaction system at 519 nm as a function of let-7a concentration (from 50 fM to 500 nM). The present method showed a linear relationship between the signal-to-noise ratio of the signal intensity of the fluorescence emission wavelength of the system at 519 nm and the logarithm of the concentration of let-7a in the range of 50 fM to 5 nM, with the linear equation F/F_0_ = 0.09944 LgC_let-7a_ + 1.204 and R^2^ = 0.9899 (Figure 4c). The detection limit was defined as the target concentration of a signal three times stronger than the standard deviation of the fluorescence intensity of the blank sample. The LOD for the detection of let-7a by this method was calculated to be 19.35 fM. Thus, the Exo III-GO-RCA strategy quantified let-7a at low concentrations, which has great potential in diagnostic assays.

**Figure 5:**
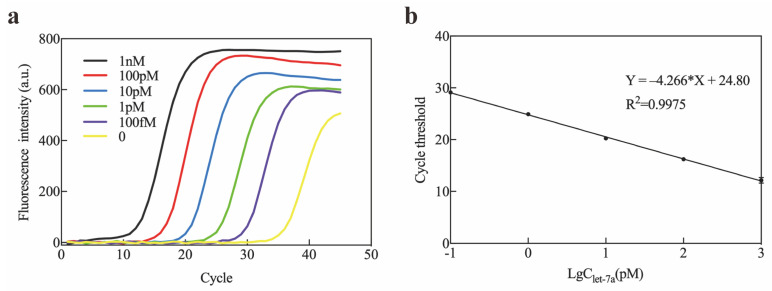
Real-time PCR amplification of let-7a: (**a**) Real-time PCR amplification profiles of let-7a; (**b**) A linear relationship was observed over the concentration range of let-7a from 100 fM to 1 nM using the RT-qPCR method in healthy human serum samples.

Considering the potential application of Exo III-GO-RCA in biological samples, this study demonstrated let-7a sequence detection in human serum. The reproducibility of the Exo III-GO-RCA platform was assessed by detecting let-7a at three different concentrations (100 pM, 1 pM, and 100 fM), and the relative standard deviations (RSDs) of the three groups were calculated separately. We added 100 pM, 1 pM, and 100 fM of let-7a standards in 100-fold diluted healthy human serum for amplification and calculated the recovery rate based on the amplification results to assess the system’s accuracy. The results are shown in Table 1. The spiked recoveries of let-7a in actual serum samples were in the range of 95% to 102.4%, and the RSDs were all lower than 5%. The experimental results indicated that the present assay had good accuracy and reproducibility, and it could be used for the detection of let-7a in actual blood samples. We further investigated the detection of let-7a by qRT-PCR. A linear relationship between let-7a concentration and cycle threshold was demonstrated (Figure 5). We found that the detection range between conventional RT-qPCR and the Exo III-GO-RCA method was comparable, and the Exo III-GO-RCA assay was superior to qRT-PCR for the quantitative detection of target RNA at low concentrations (less than 10 nM). This result indicated that miRNA could be quantitatively detected by the Exo III-GO-RCA assay with high sensitivity.

The sensitivity of the Exo III-GO-RCA method was comparable and/or even superior to that of previous detection methods (see Table 2). Due to the low detection limit, ultra-sensitive and precise quantification of miRNAs at low concentrations was possible, which is important for the early diagnosis of diseases.

### 2.4. Specificity of Exo III-GO-RCA

**Figure 6:**
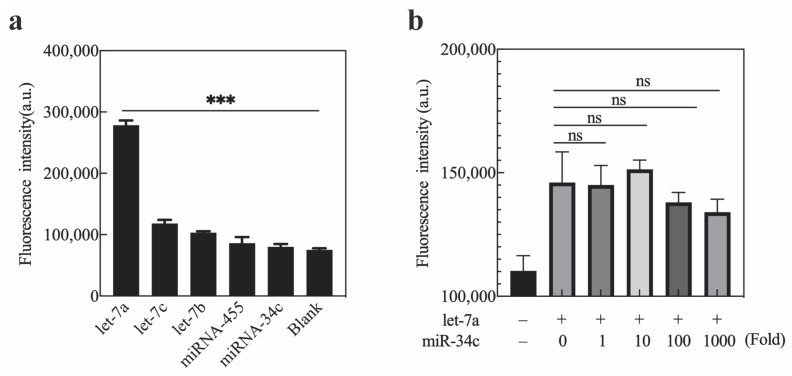
Specificity of the Exo III-GO-RCA assay: (**a**) Fluorescence intensity of the system in the presence of let-7a, let-7c, let-7b, miR-455, and miR-34c; (**b**) Fluorescence intensity of the system in the presence of let-7a with or without 1–1000-fold excess of non-target miR-34c. The excitation/emission wavelengths were 495/519 nm. Error bars reflect three separate measurements. *** *p* < 0.001. The blank group was significant, while ns means no significant difference compared with the non-target group.

To evaluate the specificity of this assay platform, we used different targets, such as let-7c and let-7b of the let-7a family, as well as non-homologous miR-455 and miR-34a. Then, we tested the specificity of the Exo III-RCA-GO platform against let-7a by detecting the fluorescence intensity values of the reaction system under each miRNA. Under the same experimental conditions, the fluorescence signal-to-noise ratios under different target miRNAs were compared, as shown in Figure 6a. Amplification could be triggered only in the presence of let-7a, and with the PPlet7a as a template, the system produced a strong fluorescence signal. We also found that the fluorescence intensity of the detection systems for let-7c (single base mutation) and let-7b (double base mutation) was the same as that of the amplification system for let-7a (single base mutation), while the fluorescence intensity of the amplification system for let-7b (double base mutation) was lower than that of let-7a and let-7a. The result indicated that the Exo III-GO-RCA method possessed high specificity for target miRNA determination.

Considering that non-target miRNAs may be present in the detection environment at higher concentrations than target miRNAs, this study investigated the selectivity of the Exo III-GO-RCA strategy for target let-7a in the presence of 1–1000-fold excess of non-target miR-34c. As shown in Figure 6b, there was no significant change in fluorescence intensity with the addition of non-target miR-34c to let-7a, even at over 1000-fold higher concentration compared to let-7a alone. Thus, this platform showed excellent specificity and strong anti-interference ability for the detection of miRNAs in complex biological samples.

### 2.5. Application of Exo III-GO-RCA in Real Sample Analysis

**Figure 7:**
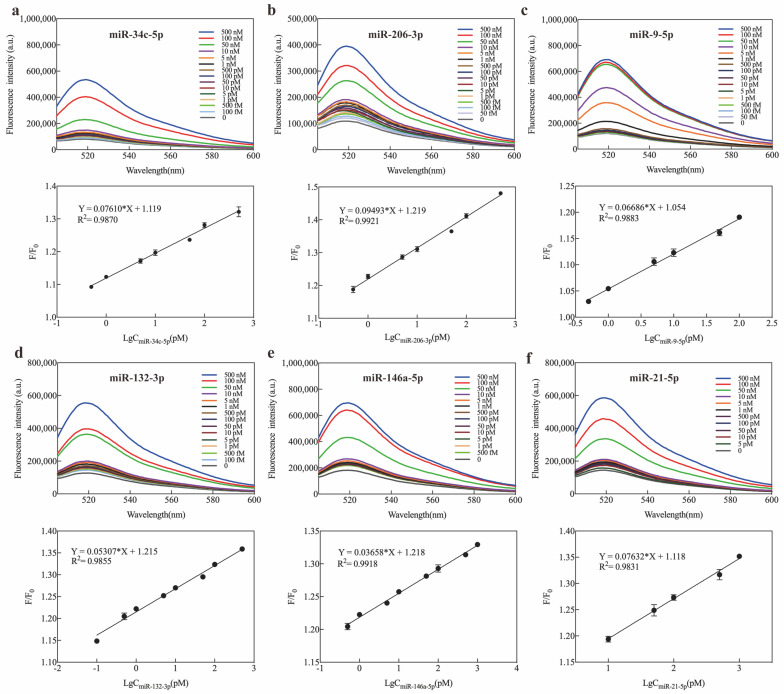
(**a**) Fluorescence response and plot of fluorescence ratio (F/F_0_) of the assay at different concentrations of miR-34c-5p; (**b**) Fluorescence response and plot of fluorescence ratio (F/F_0_) of the assay at different concentrations of miR-206-3p; (**c**) Fluorescence response and plot of fluorescence ratio (F/F_0_) of the assay at different concentrations of miR-9-5p; (**d**) Fluorescence response and plot of fluorescence ratio (F/F_0_) of the assay at different concentrations of miR-132-3p; (**e**) Fluorescence response and plot of fluorescence ratio (F/F_0_) of the assay at different concentrations of miR-146a-5p; (**f**) Fluorescence response and plot of fluorescence ratio (F/F_0_) of the assay at different concentrations of miR-21-5p. F_0_ and F are the fluorescence intensities in the absence and presence of the miRNA, respectively.

Previous works have shown the disruption of miRNA expression within AD, but miRNA regulation across the disease process has not been addressed [36,37,38,39,40]. We describe six individual miRNAs occurring frequently in AD: miR-34c-5p, miR-206-3p, miR-9-5p, miR-132-3p, miR-146a-5p, and miR-21-5p. These miRNAs exhibit inconsistent direction of dysregulation in blood and its components of AD [11,36,40,41,42,43,44,45,46,47,48]. We further evaluated the potential application of Exo III-GO-RCA to detect those miRNAs present in human serum samples. As expected, the assay showed fluorescence intensity enhancement when the concentrations of miR-34c-5p, miR-206-3p, miR-9-5p, miR-132-3p, miR-146a-5p, and miR-21-5p targets were increased using specific padlocks, respectively (Figure 7). There was a linear relationship between the signal intensity of the targets and the logarithms of the concentrations from 500 fM to 500 pM for miR-34c-5p, miR-206-3p, and miR-132-3p; 1 pM to 100 pM for miR-9-5p; 1 pM to 1 nM for miR-146a-5p; and 10 pM to 1 nM for miR-21-5p. The LOD values were 45.29 fM for miR-34c-5p, 59.04 fM for miR-206-3p, 102.9 fM for miR-132-3p, 10.05 pM for miR-9-5p, 25.3 aM for miR-146a-5p, and 521.93 fM for miR-21-5p. These results indicated that the established Exo III-GO-RCA method has great potential for routine preliminary detection of miRNAs.

### 2.6. Application of Exo III-GO-RCA Detection Platform in Early Diagnosis of APP/PS1 Mice

Studies with animal models have shown that the deposition of β-amyloid plaques in the brain of model mice is inversely correlated with SIRT1 levels [49]. Therefore, we chose miR-34c, miR-206-3p, miR-9-5p, and miR-132-3p, which target SIRT1 and regulate Aβ production [41,44,50,51,52,53], and Aβ-regulated miR-146a-5p and miR-21-5p [54,55,56,57] as the detection targets. Then, we examined the expression levels in the plasma of six APP/PS1 female and male mice from 9 to 23 weeks of age, respectively. Six C57BL/6J female and six C57BL/6J male mice served as the normal control group to explore the potential of these miRNAs as diagnostic markers for early AD diagnosis. The potential of these miRNAs as diagnostic markers in the early diagnosis of AD was explored using Exo III-GO-RCA.

**Figure 8:**
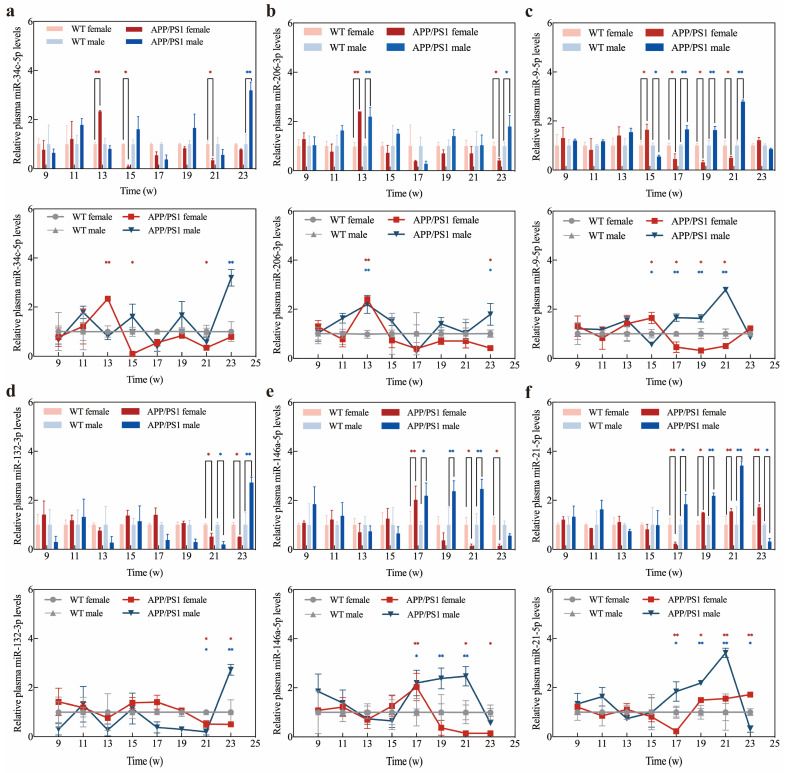
APP/PS1 mice plasma sample detection by Exo III-GO-RCA assay: (**a**) Expression levels and changes of miR-34c-5p in plasma of APP/PS1 mice from 9 w to 23 w compared to WT mice; (**b**) Expression levels and changes of miR-206-3p in plasma of APP/PS1 mice from 9 w to 23 w compared to WT mice; (**c**) Expression levels and changes of miR-9-5p in plasma of APP/PS1 mice from 9 w to 23 w compared to WT mice; (**d**) Expression levels and changes of miR-132-3p in plasma of APP/PS1 mice from 9 w to 23 w compared to WT mice; (**e**) Expression levels and changes of miR-146a-5p in plasma of APP/PS1 mice from 9 w to 23 w compared to WT mice; (**f**) Expression levels and changes of miR-21-5p in plasma of APP/PS1 mice from 9 w to 23 w compared to WT mice. * *p* < 0.05, ** *p* < 0.01 (*n* = 6 in WT group, *n* = 6 in APP/PS1 group).

In contrast to WT female mice, the expression levels of miR-34c-5p, miR-206-3p, and miR-9-5p in the plasma of APP/PS1 female mice were transiently and significantly up-regulated before 15 weeks and then maintained a continuous downregulation trend (Figure 8a–c). Changes in miR-146 and miR-21-5p expression levels showed opposite trends after 17 weeks (Figure 8e,f). Compared with WT APP/PS1 male mice, the plasma miR-34c-5p, miR-206-3p and miR-132-3p expression in APP/PS1 male mice fluctuated during AD but was upregulated at 23 weeks (Figure 8a,b,d). miR-9, miR-146, and miR-21-5p expression remained consistently upregulated after week 17 and downregulated at week 23 (Figure 8c,e,f).

**Figure 9:**
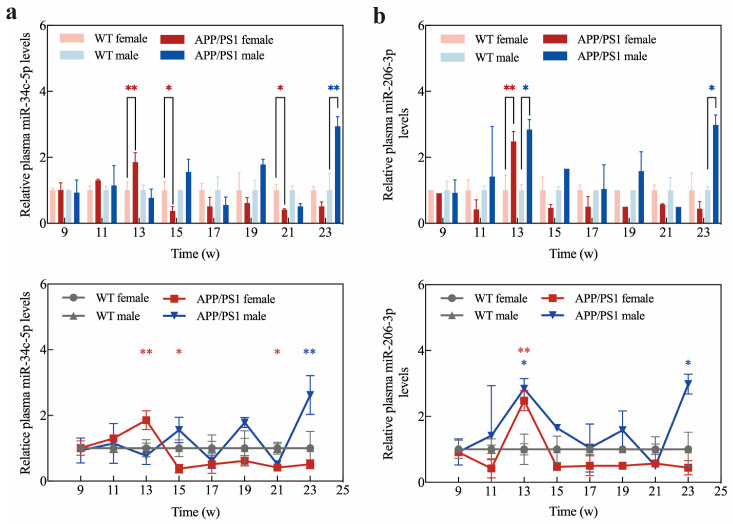
APP/PS1 mice plasma sample detection by qRT-PCR: (**a**) miR-34c-5p and miR-206-3p expression levels in plasma of WT and APP/PS1 mice; (**b**) Changes in expression levels of miR-34c-5p and miR-206-3p in plasma of APP/PS1 mice from 9 w to 23 w compared to WT mice. * *p* < 0.05, ** *p* < 0.01 (*n* = 6 in WT group, *n* = 6 in APP/PS1 group).

Furthermore, to determine the accuracy of Exo III-GO-RCA for plasma miRNA expression in APP/PS1 mice, we detected the signals of plasma miR-34c and miR-206 by qRT-PCR. The assay results are shown in Figure 9. Compared with conventional qRT-PCR, there were no significant differences in the detection of trends in plasma miR-34c and miR-206 expression levels by Exo III-GO-RCA (Figure 8a,b). Moreover, the Exo III-GO-RCA strategy was more effective in detecting miR-206-3p expression in the plasma of female mice at week 23, showing statistical differences, which was more sensitive than the qRT-PCR assay, and it can be concluded that the Exo III-GO-RCA is highly promising as an miRNA detection method for plasma samples.

## 3. Discussion

Recent clinical trials indicate that earlier intervention and diagnosis may be critical for successful AD treatment. The accurate diagnosis and timely intervention in the early stages of the disease are urgently needed. AD screening techniques are becoming reliant on molecular imaging, but early blood screening is also needed to improve the accuracy of early AD diagnosis. The continuous changes in miRNA expression levels during the progression of AD have led to conflicting research results, so there are currently no reliable miRNA diagnostic markers. Therefore, studying the trend of miRNAs expression level changes during the entire course of AD helps to find more valuable miRNA biomarkers. On the other hand, the commonly used miRNA detection methods have limitations, so the development of efficient, sensitive and highly specific detection methods is important for the diagnosis and research of AD. 

Our Exo III-GO-RCA technology was developed to detect the earliest miRNA level alterations occurring in APP/PS1 mice from 9weeks to 23weeks. Studies on the pathogenesis of AD have found that Silent Information Regulator 2 homologous protein (SIRT1) regulates the expression of Aβ [58] and Tau [59] in AD. Serum SIRT1 levels in AD patients and MCI patients are significantly lower compared to that in healthy elders of the same age [60]. Based on our results, we found that significant differences between male and female mice, and changes in the expression levels of miRNAs (miR-34c, miR-206-3p, and miR-9-5p) targeting SIRT1 and regulating Aβ production were significantly preceded by miRNAs (146a-5p and miR-21-5p) regulated by Aβ.

In the future, screening plasma samples from AD patients using our assay will find more compelling potential miRNA biomarkers for early AD diagnosis. Thus, our assay can contribute significantly to the rapid, simple screening of AD in developing countries, where molecular imaging analysis is challenging to perform. Moreover, our screening platform differs from other AD screening sensors in that it analyzes the levels of multiple miRNA AD biomarkers in plasma.

## 4. Materials and Methods

### 4.1. Materials and Apparatus

Oligonucleotides were purchased from Suzhou Genewiz Biotechnology Co., Ltd. (Suzhou, China) and were diluted to 100 μM with DEPC-treated water before use and stored in the refrigerator at −20 degrees Celsius (Haier, Qingdao, China) for spare use. phi29 DNA polymerase, dNTPs, and the Quick Genotyping Assay Kit for Mouse Tail were purchased from Beyotime Biotech Inc. (Shanghai, China); T4 DNA ligase, Exo I, Exo III, and their corresponding buffers were purchased from Takara Biomedical Technology (Beijing, China) Co., Ltd.; and GO was purchased from Nanjing Xfnano Materials Tech Co., Ltd. (Nanjing, China). All used primers can be found in Table 3. Ultrapure water was provided by a Millipore water purification system (Milli-Q, Darmstadt, Germany) and used in all experiments. A FS5 fluorescence spectrometer (Edinburgh Instruments, Edinburgh, UK) was used to detect the fluorescence of the reaction system, with excitation and emission slit widths of 3 nm, an excitation wavelength of 495 nm, and an emission wavelength of 519 nm. Human serum was purchased from Nanjing SenBeiJia Biological Technology Co., Ltd. (Nanjing, China), and the MiRcute Serum/Plasma miRNA Isolation Kit, miRcute Plus miRNA cDNA First Strand Synthesis Kit, and miRcute Plus miRNA qPCR Kit (SYBR Green) were purchased from Tiangen Biotech Co., Ltd. (Beijing, China).

### 4.2. Exo III-GO-RCA Reactions

The padlock probe (PPlet7a; 1 μM) was mixed with 1 μM of miRNA-let-7a solution, and the mixture was heated to 85 °C for 3 min and then put on ice and cooled for 15 min for annealing. Next, T4 DNA ligase (175 U) and 1 μL of 10× ligase buffer were added, the ligation mixture was incubated at 25 °C for 1 h. Subsequently, phi29 DNA polymerase (4 U) and 10× phi29 DNA polymerase buffer was added to the ligation mixture, incubated at 30 °C for 90 min, and heated at 65 °C for 10 min to terminate the polymerization reaction. For fluorescence detection, a fluorescent signaling probe (1 μM) was added to the RCA product solution, and the mixture was denatured at 85 °C for 3 min, then placed on ice and cooled for 15 min, followed by the addition of Exo III (1 U) and its reaction buffer, and the reaction was stopped by incubation at 37 °C for 15 min followed by heating at 65 °C for 5 min. GO (20 μg/mL) and 10× GO reaction buffer (50 mM MgCl_2_ and 200 mM Tris-HCl, pH 7.5) were added to the reaction solution, which was incubated at room temperature for 20 min before the luminescence signals were detected using a fluorescence spectrometer, with the parameters of the fluorescence spectrometer set to excitation at 495 nm, the slit widths of both excitation and emission were 3 nm, and the fluorescence response of the sample was measured at 505 nm to 600 nm. The luminescence signal intensity of the sample at 519 nm was used for quantitative analysis of the target miRNA.

### 4.3. Mice Plasma miRNA Extraction and qRT-PCR Detection

Mice were bred from two pairs of APP/PS1 transgenic and C57BL/6J mice purchased from Vital River Laboratory Animal Technology Co., Ltd. (Beijing, China), and the genotype of their filial mice was identified by the Quick Genotyping Assay Kit for Mouse Tail. We used *n* = 6 animals per group. Animal housing was carried out in ventilated racks at 23 °C and under a 12 h light–dark cycle. Mice plasma samples were collected from C57BL/6J mice and APP/PS1 mice every two weeks from 9 to 23 weeks of age. A 200 µL sample of blood was collected from the submandibular venous plexus, and the supernatant obtained by centrifugation at 3000 rpm for 10 min was used as the plasma sample. Mice plasma samples were subjected to miRNA extraction according to the miRcute Serum/Plasma miRNA Isolation Kit from Tiangen. cDNA samples were prepared using the miRcute Plus miRNA cDNA First Strand Synthesis Kit. The miRNA was analyzed by qRT-PCR according to the instructions of Tiangen’s miRcute Plus miRNA qPCR Kit (SYBR Green).

## 5. Conclusions

In this study, we have developed a simple, highly sensitive, and specific Exo III-GO-RCA platform for multiple miRNA detection. The platform utilizes an amplification strategy by exploiting the action of phi29 DNA polymerase and Exo III enzyme to generate a great increase in the concentration of released fluorescein in the presence of GO quencher. Our platform showed highly sensitive detection of plasma AD miRNA biomarkers at the femtomolar level. Moreover, this method had excellent specificity to discriminate between different miRNA targets or between mutant miRNA let-7a sequences differing by only one base. Importantly, the Exo III-GO-RCA assay was successfully applied to rapidly detect six miRNAs in plasma from APP/PS1 mice. Unlike conventional qRT-PCR techniques, which require cooling cycles and reverse transcription, the Exo III-GO-RCA method was performed at room and constant temperature. It also offers several advantages, such as specific recognition of target sequence, inexpensive operation, as well as shorter detection times, making it ideal for clinical diagnostic applications. Moreover, the platform was used to detect a variety of miRNAs, namely, miR-34a-5p, miR-206-3p, miR-9-5p, miR-132-3p, miR-146a-5p, and miR-21-5p. This platform did not merely analyze the levels of six miRNAs in plasma but combined consistent levels of these biomarkers. As such, this method substantially increases the accuracy of plasma-based AD screening. The consistency of changes in the expression levels of these miRNAs has the potential to screen the early stages of AD. Taken together, our study demonstrates that the established Exo III-GO-RCA method could be applied for quantitative analysis of target miRNA sequences in complex environments.

## Figures and Tables

**Table 1:** Recovery detection in healthy human serum sample.

Added (pM)	Found (pM)	Recovery (%)	RSD (±%)
100	95.5	95.5	4.28376963
1	1	100	2.939
0.1	0.1024	102.4	4.7109375

**Table 2:** Comparison of the reported strategies for miRNA detection.

Method	Target	Samples	LOD	Analysis Time	Ref.
Nicking-Enhanced RCA with MoS_2_ QDs	Let-7a	Serum	4.6 fM	5.3 h	[22]
Branched RCA	miR-21	Serum	1 pM	5 h	[25]
AuNP with CRISPR/Cas12a-assisted HRCA	miR-143	Cell, urine	100 aM	2 h	[21]
DSNSA with WS_2_ nanosheet	miR-21	Cell	300 fM	5 h	[35]
RCA integrated catalytic hairpin assembly	Let-7b	Serum	10 pM	4.3 h	[32]
Nicking-Enhanced RCA with GO	miR-21	Saliva	1.4 fM	2.4 h	[20]
Exo III-GO-RCA	Let-7a	Serum	19.35 fM	4 h	This work

**Table 3:** Sequences of oligonucleotides and miRNAs in this study.

Name	Sequence (5′–3′)	Length
let-7a-5p	UGAGGUAGUAGGUUGUAUAGUU	22 bp
PPlet7a	p-CTACTACTCACCCTAACCCTAACCCTAACCCTAACC CTAACCCTAAAACTATACAAC	58 bp
F-DNA	FAM-CCCTAACCCTAA	12 bp
let-7b	UGAGGUAGUAGGUUGUGUGGUU	22 bp
let-7c	UGAGGUAGUAGGUUGUAUGGUU	22 bp
miR-455-3p	AUGCAGUCCAUGGGCAUAUACAC	23 bp
miR-34c-5p	AGGCAGUGUAGUUAGCUGAUUGC	23 bp
miR-206-3p	UGGAAUGUAAGGAAGUGUGUGG	22 bp
miR-9-5p	UCUUUGGUUAUCUAGCUGUAUGA	23 bp
miR-132-3p	UAACAGUCUACAGCCAUGGUCG	22 bp
miR-146a-5p	UGAGAACUGAAUUCCAUGGGUU	22 bp
miR-21-5p	UAGCUUAUCAGACUGAUGUUGA	22 bp
PP34c	p-ACTACACTGCCTCCCTAACCCTAACCCTAACCCTA ACCCTAACCCTAAGCAATCAGCTA	59 bp
PP206	p-CTTACATTCCACCCTAACCCTAACCCTAACCCTAA CCCTAACCCTAACCACACACTTC	58 bp
PP9	p-GATAACCAAAGACCCTAACCCTAACCCTAACCCT AACCCTAACCCTAATCATACAGCTA	59 bp
PP132	p-GTAGACTGTTACCCTAACCCTAACCCTAACCCTA ACCCTAACCCTAACGACCATGGCT	58 bp
PP146a	p-TTCAGTTCTCACCCTAACCCTAACCCTAACCCTA ACCCTAACCCTAAAACCCATGGAA	58 bp
PP21	p-TGATAAGCTACCCTAACCCTAACCCTAACCCTAA CCCTAACCCTAATCAACATCAGTC	58 bp

## Data Availability

The original contributions presented in the study are included in the article material; further inquiries can be directed to the corresponding author.
